# Harnessing Noxa demethylation to overcome Bortezomib resistance in mantle cell lymphoma

**DOI:** 10.18632/oncotarget.2903

**Published:** 2015-02-25

**Authors:** Violetta V. Leshchenko, Pei-Yu Kuo, Zewei Jiang, Marc A. Weniger, Jessica Overbey, Kieron Dunleavy, Wyndham H. Wilson, Adrian Wiestner, Samir Parekh

**Affiliations:** ^1^ Division of Hematology and Medical Oncology, Icahn School of Medicine at Mount Sinai, New York, NY, USA; ^2^ Hematology Branch, National Heart, Lung and Blood Institute, National Institutes of Health, Bethesda, MD, USA; ^3^ Department of Health Science and Policy, Icahn School of Medicine at Mount Sinai, New York, NY, USA; ^4^ Lymphoid Malignancies Branch, Center for Cancer Research, National Cancer Institute, National Institutes of Health, Bethesda, MD, USA

**Keywords:** Mantle cell lymphoma, Noxa, methylation, Bortezomib, Resistance

## Abstract

Bortezomib (BZM) is the first proteasome inhibitor approved for relapsed Mantle Cell Lymphoma (MCL) with durable responses seen in 30%–50% of patients. Given that a large proportion of patients will not respond, BZM resistance is a significant barrier to use this agent in MCL. We hypothesized that a subset of aberrantly methylated genes may be modulating BZM response in MCL patients. Genome-wide DNA methylation analysis using a NimbleGen array platform revealed a striking promoter hypomethylation in MCL patient samples following BZM treatment. Pathway analysis of differentially methylated genes identified molecular mechanisms of cancer as a top canonical pathway enriched among hypomethylated genes in BZM treated samples. Noxa, a pro-apoptotic Bcl-2 family member essential for the cytotoxicity of BZM, was significantly hypomethylated and induced following BZM treatment. Therapeutically, we could demethylate Noxa and induce anti-lymphoma activity using BZM and the DNA methytransferase inhibitor Decitabine (DAC) and their combination *in vitro* and *in vivo* in BZM resistant MCL cells. These findings suggest a role for dynamic Noxa methylation for the therapeutic benefit of BZM. Potent and synergistic cytotoxicity between BZM and DAC *in vitro* and *in vivo* supports a strategy for using epigenetic priming to overcome BZM resistance in relapsed MCL patients.

## INTRODUCTION

Mantle Cell Lymphoma (MCL) is an aggressive and mostly incurable B cell malignancy with frequent relapses after initial response to standard chemotherapy. Bortezomib (BZM) is the first proteasome inhibitor approved by the FDA for relapsed and refractory MCL, with durable responses in 30%–50% of MCL patients in phase II studies [1, 2]. BZM is a highly selective, reversible inhibitor of 26S proteasome leading to the modulation of several biological processes, such as cell cycle arrest, induction of apoptosis, deregulation of NF-κB activity, and induction of endoplasmic reticular (ER) stress. However, additional mechanisms contributing to its cytotoxicity continue to be characterized. Our group and others have identified aberrantly methylated genes in MCL controlling critical cellular processes like cell cycle, transcription and regulation of gene expression [3]. Here, we hypothesized that a subset of aberrantly methylated genes may be contributing to the response to BZM treatment by inducing DNA hypomethylation and cellular reprogramming leading to a significant antitumor activity.

The cytotoxicity of BZM in primary MCL, multiple myeloma and other neoplastic cells has been associated with pro-apoptotic phorbol-12-myristate-13-acetate-induced protein 1 (PMAIP1/Noxa) expression [4]. Noxa accumulation antagonizes the function of anti-apoptotic Bcl-2 family members and results in apoptotic cell death. Noxa is regulated in a stimulus dependent manner by various transcription factors including p53, HIF1α and c-MYC [4]. It is currently unknown whether modulation of Noxa gene methylation contributes to Noxa activation in MCL following BZM-based therapy.

Here we investigate the relationship between BZM treatment and Noxa methylation in MCL patient samples. Furthermore, we investigate whether modulation of Noxa methylation using BZM and the DNA methytransferase inhibitor Decitabine (DAC) can overcome BZM resistance *in vitro* and in xenograft models.

## RESULTS

### Proteosome inhibitor BZM causes global DNA hypomethylation including Noxa and other Bcl-2 family members in tumor cells from MCL patients

In order to examine the genomic methylation changes after BZM treatment, we used the HpaII tiny fragment Enrichment by Ligation–mediated PCR (HELP) assay that covers more than 25,626 CpGs over 14,000 gene promoter regions [5]. Genomic DNA was extracted from tumor cells purified from the peripheral blood of 6 newly diagnosed MCL patients treated with single-agent BZM at the National Institutes of Health. Matched samples were obtained at baseline and at 96 hours after treatment start.

**Figure 1:**
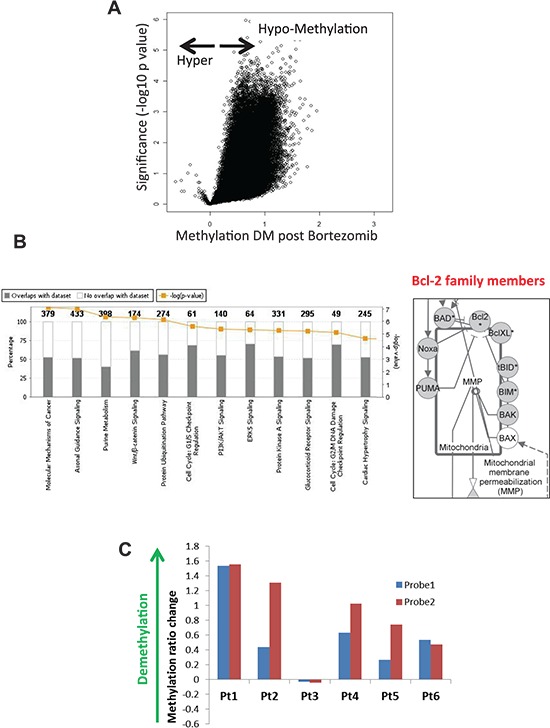
Proteasome inhibitor BZM causes global DNA hypomethylation including Noxa and other Bcl-2 family members in tumor cells from MCL patients **(A)** Volcano plot showing difference of mean methylation in MCL patient samples after BZM treatment (X axis) vs. significance (Y axis) shows predominant genomic hypomethylation in DNA from tumor cells from MCL patients following treatment with single agent BZM. **(B)** *Right* - Ingenuity Pathway Core Analysis of the differentially methylated genes after BZM treatment of MCL patients. *Left* - Bcl-2 family members among differentially methylated genes after BZM treatment. Differentially methylated loci (*p* < 0.05, DM > 0.5) in patients with MCL are indicated in grey (hypomethylated). **(C)** Changes in methylation values estimated by HpaII/MspI ratios difference (Y-axis) in two Noxa promoter HELP array probes in MCL patient samples after treatment with BZM using HELP array.

All the methylation array datasets passed a rigorous quality control and quantile normalization procedure. Analysis of methylation revealed a striking genome-wide hypomethylation following BZM as shown in Figure 1A. These findings were confirmed independently using the MethylFlash Methylated DNA Quantification colorimetric assay for 5-methylcytosine-modified genomic DNA (Supplementary Figure 1A). Interestingly, we observed a significant reduction in DNMT1 levels following BZM treatment in MCL cells (Supplementary Figure 1B) similar to the results on AML cells reporting BZM as a potent inhibitor of DNA methylation [6].

13250 differentially methylated loci (*p*-value < 0.05, DM > 0.5), which correspond to 13102 unique Refseq IDs and to 9561 annotated genes were hypomethylated after BZM treatment (Supplementary Table 1). Gene Set Enrichment Analysis (GSEA) was used to categorize members of a gene set by gene families with transcription factors representing the largest gene family (Supplementary Table 2). Pathway analysis of differentially methylated genes after BZM treatment revealed molecular mechanisms of cancer (*p*-value = 8.50E-08, Ratio = 0.522 [198/379]), protein ubiquitination, and cell cycle regulation pathways as the top canonical pathways (Figure 1B, Supplementary Table 3) and gene expression, cell growth and proliferation, and cell death and survival as the top molecular functions (Supplementary Table 4). Among the genes contained in the molecular mechanisms of cancer pathway are the members of the Bcl-2 family. These important regulators of the mitochondrial apoptosis pathway include the pro-apoptotic members *PMAIP1/Noxa, BCL2L11/Bim, BAD, BID*, *Puma*, and *Bak,* all of which were hypomethylated after BZM treatment (Figure 1B, Supplementary Table 5). Pro-apoptotic gene *BIM* is frequently deleted and BIM protein expression is absent in most MCL as previously published [7]. Noxa induction after BZM treatment is critical for BZM-induced cytotoxicity specifically in primary MCL *in vivo* and *in vitro* [8]. Other BH3-only proteins were not affected by BZM exposure in MCL cell line models as previously reported [9]. In our experiments, demethylation of the Noxa promoter in the region covered by two probes designed in HELP array was observed in 5 out of 6 patient samples after treatment with BZM (Figure 1C).

### Noxa can be therapeutically demethylated and induced by BZM and DAC in MCL cell lines

**Figure 2:**
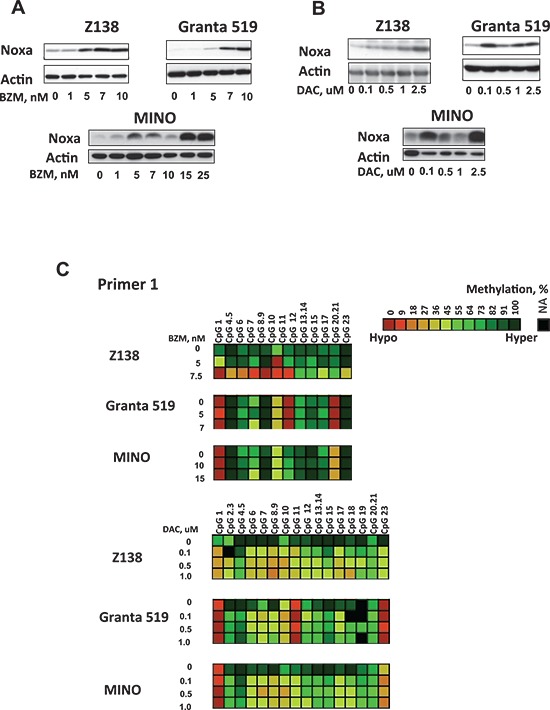
Noxa can be therapeutically demethylated and induced by BZM and DAC in MCL cell lines **(A–B)** Western blots were done after treatment of MCL cells with single doses of BZM for 48 hours or three sequential daily doses of DAC for 72 hours. **(C)** MassArray confirms Noxa demethylation in Z138, Granta 519, and MINO cells after treatment with DAC and BZM. Scale shows percentage of methylation from 0% (low, in dark orange) to 100% (high, in dark green) for each CpGs.

After demonstrating Noxa demethylation in MCL patient samples following BZM treatment, we wanted to understand whether Noxa demethylation induced Noxa gene expression and caused cytotoxicity in MCL. In our experiments, BZM decreased cell viability in the dose range of 1–25 nM (Supplementary Figure 2A) in six MCL cell lines examined. MCL cell lines showed a bimodal pattern of response to BZM with 3 BZM-sensitive (Z138, Granta 519, JeKo-1) and 3 BZM-resistant cell lines (MINO, Rec-1, NCEB-1) (Supplementary Figure 2A), as previously published [9]. We observed a dose-dependent Noxa induction 24 hours after BZM treatment in five MCL cell lines (Figure 2A, Supplementary Figure 2B). Next, we evaluated the efficacy of DAC, a well-characterized DNA hypomethylating agent, in the induction of Noxa protein in a range of concentrations achievable in human plasma [10]. Previously we have reported that a multi-day sequential schedule of treatment with DAC is more efficient for global demethylation in MCL cell lines [3]. We found that DAC treatment causes a dose-dependent increase in Noxa protein level in a set of MCL cell lines (Figure 2B). HELP array analysis confirmed demethylation of Noxa promoter in two MCL cell lines MINO and Z138 after DAC treatment (Supplementary Figures 3A and 3B). HELP array findings were validated using the Sequenom MassArray platform, which allows analysis of methylation at the individual CpG sites (Figure 2C). Each HELP probe covers the flanking HpaII sites for a given HpaII amplifiable fragment (HAF), as well as any other HpaII sites found up to 2,000 bp upstream of the downstream site and up to 2,000 bp downstream of the upstream site. Five MassArray primers spanning 136 individual CpGs were constructed to cover two Noxa HELP array probes and a CpG island located in the Noxa promoter close to the transcriptional start site (TSS) of the Noxa gene (Supplementary Figure 3A). In agreement with the array data, a difference in DNA methylation status of the Noxa promoter (30–50%) was evident between untreated and both BZM and DAC-treated cells in CpGs covered by primers 1 and 2 corresponding to nucleotides −1429 to −941 and −967 to −512 relative to TSS, respectively (Figure 2C, Supplementary Figure 3C). There were no changes seen in the methylation of CpGs covered by primers 3, 4, and 5 corresponding to nucleotides −426 to +975 and located within CpG island in the promoter region of the PMAIP1 gene in MCL cell samples 24 hours after BZM treatment and 72 hours after DAC treatment (data not shown). Notably, the pattern of CpG methylation was different following BZM and DAC treatment, suggesting that BZM and DAC may affect different CpG dinucleotides within Noxa promoter. For the area covered by primer 1, the majority (20–22 sites; 86–100%) of the sites were completely or partially methylated in cells (complete methylation; 80–100%, partial methylation; 20–60% and complete demethylation; 0%) before any treatment in three MCL cell lines. BZM treatment caused demethylation of CpG sites 4 through 11 mostly in the BZM-sensitive Z138 cell line, whereas DAC affected the methylation status more broadly by demethylating 15 CpGs in total (Figure 2C). DAC in the concentration range tested (0–1uM) displayed 15–20% cytotoxicity in 3 MCL cell lines (Supplementary Figure 3D).

### DAC sensitizes MCL cell lines to BZM via Noxa induction

**Figure 3:**
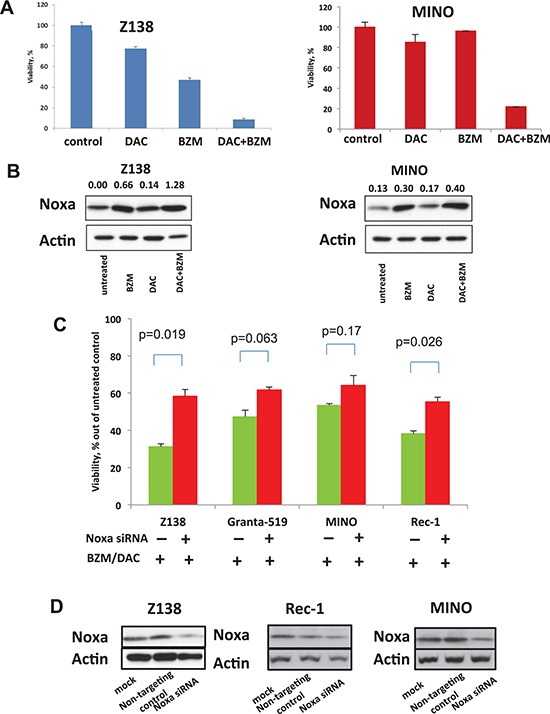
DAC sensitizes MCL cell lines to BZM via Noxa induction **(A)** *Left* - Low dose (0.5 μM) DAC pre-treatment potentiated IC50 dose BZM causing more than 80–90% cell kill in Z138 cells. *Right* - Low dose (0.1 μM) DAC pre-treatment potentiated IC25 (10 nM) dose BZM causing more than 80% cell kill in MINO cells. **(B)** Western blot of Noxa protein expression after Z138 and MINO cell line treatment with BZM, DAC and their combination. **(C)** Depletion of Noxa by specific shRNA rescued Z138, Granta 519, MINO, and Rec-1 cells from cytotoxicity of BZM and DAC combination. MCL cells were transfected with non-targeting siRNA (non-targeting control) and with Noxa siRNA and treated 24 hours after transfection with the following combinations: 7 nM BZM and 0.1 μM DAC for Z138; 7 nM BZM and 1 μM DAC for Granta 519; 15 nM BZM and 1 μM DAC for MINO, and 25 nM BZM and 1 μM DAC for Rec-1. DAC was given as three sequential daily doses indicated above. Viability was assessed by trypan blue exclusion 72 hours after treatment. The results showed are the mean of 2 different experiments. Data were analyzed using the Student's *t*-test. **(D)** Noxa protein expression in MCL cells following Noxa depletion and detected by Western blotting analysis 24 hours after transfection.

We then tested whether epigenetic priming by DAC could sensitize cells to BZM cytotoxicity in a Noxa-dependent manner. We pre-treated the BZM-sensitive Z138 cells and BZM-resistant MINO cells with non DNA damaging doses of DAC in the range of 0.05 μM to 0.5 μM for 72 hours followed by BZM at the concentrations below IC_50_ for another 48 hours. Pre-treatment of BZM-sensitive Z138 cell line with low doses of DAC (0.5 μM) followed by 7.5 nM of BZM resulted in 90% cell kill (Figure 3A). For MINO cells, BZM and DAC alone at single doses killed about 10–25% of cell while drug combination killed around 80% of cells as shown on the viability plot (Figure 3A). The combination index (CI) values for synergy assessed by the method of the Chou-Talalay [11] for DAC and BZM at concentrations achievable in human plasma were well below 1 cutoff for Z138 and MINO cells representing a strong synergistic effect of the two drugs (Supplementary Figures 4A and 4B). Interestingly, the CI values between DAC and BZM combinations were lower in MINO as compared to Z138 cells indicating more synergy between these drugs for the BZM-resistant cell line (Supplementary Figures 4A and 4B). Notably, the combination of the two drugs led to an increase in Noxa protein level as compare to single drug treatment in both MINO and Z138 cells (Figure 3B). The synergistic effect of DAC and BZM was observed for both treatment schedules when we pre-treated cells with DAC as well as when the two drugs were simultaneously added to the cells. The synergistic effect of the two drugs was observed in two more BZM-resistant MCL cell lines Rec-1 and NCEB-1 displaying the therapeutic potential of the combination (Supplementary Figures 4C and 4D).

To understand the specific contribution of Noxa to BZM and DAC-induced cytotoxicity, we used small interfering RNA (siRNA) to suppress the expression of Noxa. We first transfected cells with Noxa siRNA. Then the cells were treated with BZM and DAC. We found that specific depletion of Noxa by siRNA abrogates cytotoxicity from the BZM and DAC combination in all four MCL cell lines tested (Figure 3C). Noxa protein depletion was confirmed by western blotting 24 hours post transfection (Figure 3D).

### BZM and DAC synergize *in vivo* in MCL xenograft models

**Figure 4:**
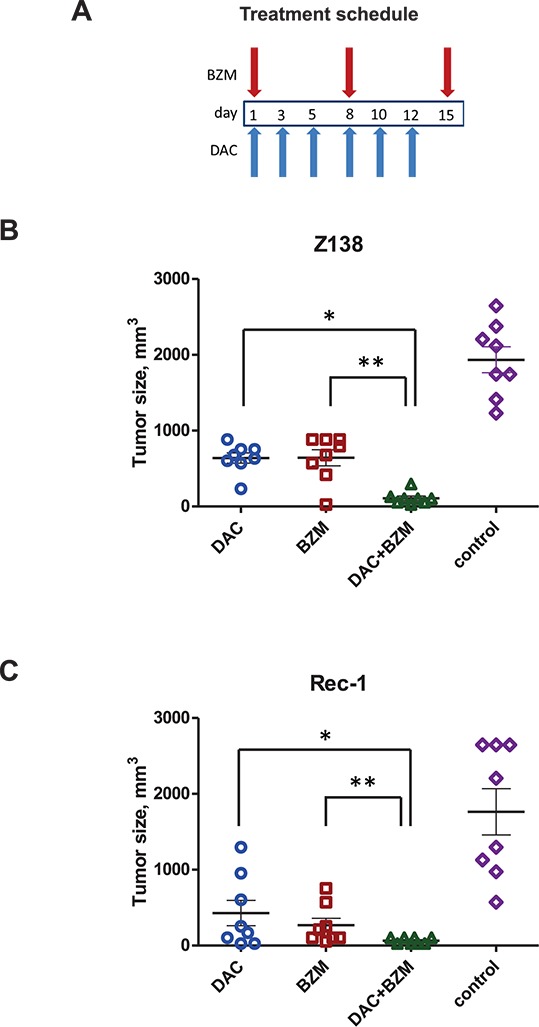
BZM and DAC synergize in the MCL xenograft models **(A)** Treatment schedules for groups of animals receiving both DAC and BZM. **(B–C)** Z138 and Rec-1 MCL cell lines were used. Tumor volumes for treatment cohorts expressed as means +/– SEM in 16 days from the beginning of treatment. DAC was given IP as 6 injections of 0.2 mg/kg on day 1, 3, 5, 8, 10, 12 (1.2 mg/kg total), BZM was injected SC, 1 injection of 75 μg/kg (Z138) or 100 μg/kg (Rec-1) per week during 3 weeks as shown on treatment schedule schemes. Control group of mice received SC diluent control (0.01% DMSO) injection once per week during 3 weeks. *N* = 8 for each group of mice. Statistical significance between the groups is indicated as follows: for Z138 xenografts *P < 0.000005 for DAC+BZM vs DAC alone; **P < 0.003 for DAC+BZM vs BZM alone; for Rec-1 xenografts *P < 0.05 for DAC+BZM vs DAC alone; **P < 0.05 for DAC+BZM vs BZM alone.

To determine whether our *in vitro* finding could be translated to *in vivo* setting, we examined the effect of epigenetic priming by DAC to BZM cytotoxicity using xenografts models of the BZM-resistant MCL cell line Rec-1 as well as the BZM-sensitive cell line Z138. For each cell line xenograft experiment, immunocompromised athymic Nude-*Foxn1^nu^* mice were treated with individual drugs as well as the combination of BZM and DAC according to the schedule indicated on Figure 4A. In MCL xenograft models, tumors are expected to become palpable 2–3 weeks post injection. For the Z138 xenograft model, statistically significant tumor growth inhibition was observed in the drug combination group of animals comparing to control group (*P* < 0.0000001) in 16 days from the beginning of treatment as well as to single agent treatment with DAC alone (*P* < 0.000005) or BZM alone (*P* < 0.003) (Figure 4B). Both of the single drug groups had a statistically significant tumor growth inhibition compared to the control group (*P* < 0.00003). Similarly, for the BZM-resistant cell line Rec-1, the combination of BZM and DAC caused further statistically significant tumor reduction as compared to the control group of mice (*P* < 0.001) as well as single agent cohorts (*P* < 0.05), illustrating the potency of this combination in BZM resistant MCL (Figure 4C). We did not observe significant weight loss (i.e. > 10%) in any of animal groups for both Z138 and Rec-1 xenograft models (Supplementary Figures 5A and 5B). We then tested one more BZM-resistant MCL cell line MINO *in vivo*. Our results with MINO show significant tumor shrinkage with single agents comparing to vehicle control treatment (*P* < 0.005) after 16 days of injection (Supplementary Figure 5C). The combination therapy caused more effective decrease in tumor as compare to control (*P* < 0.001) although not statistically significant as compare to single drugs (Supplementary Figure 5C). Both drugs and their combination were well tolerated by the mice and were not accompanied with weight loss in any of animal groups in MINO xenograft models (Supplementary Figure 5D). Taken together, our results indicate that BZM and DAC treatment had a similar synergistic effect in MCL cells *in vivo* as that observed *in vitro*.

## DISCUSSION

The present data show the induction of genome-wide hypomethylation in MCL patient samples following BZM treatment, suggesting that the BZM induced epigenetic changes may contribute to its cytotoxic mechanism in MCL.

In AML models, BZM has been reported as a potent inhibitor of DNA methylation in malignant cells by interfering with Sp1/NF-κB DNA–binding activity, which in turn results in decreased DNMT1 expression, DNA hypomethylation, and transcription of methylation-silenced genes [6]. Our findings on global DNA hypomethylation following BZM treatment are in concordance supporting BZM as a novel, non-azanucleoside therapeutic agent to target aberrant DNA hypermethylation in cancer. We observed a significant reduction in DNMT1 levels following BZM treatment in MCL cells similar to the results reported in AML cells (Supplementary Figure 1B).

The Bcl-2 homology3 (BH3)-only pro-apoptotic protein Noxa contributes to apoptosis in response to genotoxic agents, cyclin-dependent kinase inhibitors, histone deacetylase inhibitors, or inhibitors of the 26S proteasome such as BZM [4]. Noxa can be induced in both a p53-dependent and independent fashion in response to cellular stress [4]. In MCL models, Noxa transcription is activated by two cooperating mechanisms, the induction of transcriptional factors ATF3 and ATF4 independently of p53 and blockade of histone H2A ubiquitination [12]. Little is known about epigenetic alterations in *Noxa* methylation in response to chemotherapy. In memory T-cells, the Polycomb group (PcG) gene Bmi1 controls cell survival by directly repressing Noxa gene expression. Bmi1 is required for DNA CpG methylation of the Noxa gene and for binding of DNMT1 and other PcG gene products to its promoter [13]. Recently, MiR-200c, a miRNA highly expressed in MCL and epigenetically controlled in both normal and cancer cells, was shown to repress both basal and stress-induced Noxa protein expression [14, 15] suggesting additional epigenetic contribution to the regulation of Noxa expression. Our results show the induction of hypomethylation of the *Noxa* promoter in response to BZM treatment. We identified and validated a set of CpG sites within the Noxa promoter region associated with Noxa expression after BZM and demethylating agent DAC treatment and confirmed DNA hypomethylation after treatment (Figure 2C). Recent studies reported that cAMP response element binding protein (CREB) is involved in the transcriptional induction of Noxa [16]. CpGs demethylated by BZM and DAC, as shown by our MassArray data, are located in the Noxa promoter region and are close to CREB binding sites.

Despite recent advances in the treatment of MCL with BZM, not all patients respond, and resistance often appears after initial treatment. In MCL cell lines, BZM resistance is associated with plasmacytic differentiation, which in the absence of an increased secretory load can enable cells to withstand the stress of proteasome inhibition [17]. In multiple myeloma models, BZM therapy induces quiescence and survival of residual MM cells, contributing to disease recurrence [18]. Here we show that epigenetic priming by DAC can be a novel therapeutic strategy for overcoming BZM resistance in MCL. This may be a promising approach in suppression of survival/adaptation responses such as tumor cell quiescence, an undesirable side effect of proteasome inhibition [18]. Our data suggest that demethylation of the Noxa promoter by BZM and DAC may be a key modulator of Noxa activation in BZM-induced cell death in MCL. More broadly, our data demonstrate that genomic methylation profiling can help us identify novel drug mechanisms and therapeutic combinations in MCL.

## MATERIALS AND METHODS

### Patient samples

Peripheral blood samples were obtained from 6 patients newly diagnosed with MCL leukemic disease before any treatment and 96 hours after BZM treatment at the National Cancer Institute. Sample collection and laboratory studies were in compliance with institutional review board and Helsinki protocols. Peripheral blood mononuclear cells were separated by gradient centrifugation using Lymphocyte Separation Medium (MP Biomedicals) and CD19+ B cells were purified by magnetic-activated cell sorting using CD19 microbeads (Miltenyi Biotec) to ensure greater than 90% purity for HpaII tiny fragment Enrichment by Ligation–mediated polymerase chain reaction (HELP PCR) analysis.

### Cell lines, culture conditions, and drug treatment

MCL cell lines Granta 519, JeKo-1, MINO, NCEB-1, Rec-1, and Z138 were cultured in RPMI 1640 medium (Cellgro) supplemented with 10% fetal bovine serum (FBS; Gemini Bio-Products), 100 U/mL penicillin G, and 100 g/mL streptomycin (Cellgro), at 37C with humidification. Cells were obtained from the American Type Culture Collection. BZM was purchased from Selleck Chemicals and formulated at stock solutions at 50uM after dissolution in DMSO. DAC was obtained from Sigma Aldrich and formulated at 1mM. All drugs were stored at between −20 and −80C. BZM and DAC were used in the therapeutically relevant range of concentrations (BZM 1–25 nM, DAC 0.05–2.5 μM) [10, 19]. Cells were treated in series of eight 100 μl wells for 48 hours for viability assessment and in 3 ml wells in triplicate for 24 or 72 hours for siRNA knockout experiments and to determine mRNA level and protein amounts. Sequences for siRNA knockout are indicated in Supplementary Table 6.

### Cell viability assay and assessment of synergy

Cell viability was determined by a fluorometric resazurin reduction method (CellTiter-Blue; Promega) following the manufacturer's instructions. The number of viable cells in each treated well was calculated 48 hours after treatment. Cells (100 μL; 10^5^ cells per well) were plated in 96-well plates (8 replicates per condition), with 20 μL of CellTiter-Blue Reagent (Promega) added to each well. After 1 hour of incubation with the dye, fluorescence (560_Ex_/590_Em_) was measured with the FLUOstar Omega microplate reader (BMG Lab Technologies). The number of viable cells in each treated well was calculated, based on the linear least-squares regression of the standard curve. Cell viability in drug-treated cells was normalized to their respective untreated controls. Cell counts were confirmed on the Countess automated cell counter (Invitrogen) according to the manufacturer's specifications. Data were analyzed using the Student's *t*-test. Synergistic effect of drug-drug interaction was evaluated by Chou and Talalay median effects analysis [11] using Calcusyn Software (Biosoft). Degrees of synergism are expressed as combination indices (CI), with smallest values indicating the most synergy. CI values < 0.8 indicate synergy; those 0.8–1.2 indicate an additive effect; and those > 1.2 indicate antagonism.

### DNA methylation analysis by HELP

Genomic DNA was isolated using a standard high-salt procedure. HELP assay, a comparative isoschizomer profiling method interrogating cytosine methylation status on a genomic scale, was carried out as previously described [5, 20]. Briefly, genomic DNA from the samples was digested by a methylcytosine-sensitive enzyme, HpaII, in parallel with MspI, a methylcytosine-insensitive enzyme. The HpaII and MspI digested products were amplified by ligation-mediated PCR. PCR condition has been optimized to amplify fragments between 200 and 2000 base pair (bp), ensuring the preferential amplification of cytosine-phosphate-guanosine (CpG) dinucleotide-dense regions. Each fraction is then labeled with a specific dye and cohybridized onto a human HG17 custom-designed oligonucleotide array (50-mers) covering 25626 HpaII amplifiable fragments (HAFs) located at gene promoters and imprinted regions across the genome [20]. HAFs are defined as genomic sequences between 2 flanking HpaII sites found within 200 to 2000 bp from each other. Each HAF on the array is represented by 15 individual probes. DNA methylation was measured as the log (HpaII/MspI) ratio, ranging from −4.0 to 6.0, where negative values indicate higher levels of cytosine methylation and vice versa. All samples for microarray hybridization were processed at the Roche-NimbleGen Service Laboratory. Scanning was performed with the use of a GenePix 4000B scanner (Axon Instruments). PCR fragment length bias was corrected by quantile normalization. Further quality control and data analysis of HELP microarrays were performed as described in Thompson and colleagues [21]. Microarray data sets are available from the Gene Expression Omnibus (GEO) repository (GSE58165).

### Global DNA methylation analysis

The amount of global, genome-wide DNA methylation was quantified using the Methylamp global DNA methylation quantification kit (Epigentek) according to the manufacturer's instructions. In this assay, 5-methylcytosine-modified genomic DNA is recognized by 5-methylcytosine antibody, and the bound DNA is quantified in a fluorometric assay. Positive (methylated) and negative (unmethylated) control DNA was supplied with the kit. Fluorescence was measured on the FLUOstar Omega microplate reader (BMG Lab Technologies). The amount of DNA methylation (percent methylation) was calculated using the following formula: percent methylation = [OD (sample − negative control) × GC content]/[OD (positive control − negative control) × 10] × 100%.

### Quantitative DNA methylation analysis by massARRAY epityper

Validation of HELP findings was performed by Matrix-Assisted Laser Desorption Ionization Time-Of-Flight (MALDI-TOF) mass spectrometry by MassARRAY (Sequenom) as previously described [3, 22]. With the Sequenom EpiDesigner program, 5 primer sets were generated to analyze methylation of Noxa promoter. Briefly, PCR primers specific for bisulfite-converted genomic DNA were designed to cover the flanking HpaII sites for a given HAF, as well as any other HpaII sites found up to 2,000 bp upstream of the downstream site and up to 2,000 bp downstream of the upstream site. Primer sequences indicated in Supplementary Table 6.

### Microarray data analysis and gene network analysis

The final set of candidates was defined as those genes differentially methylated between post- and pre-BZM treated patient samples with a *p*-value below 0.05 and differences of mean above 0.5. This cutoff was chosen in order to provide a reasonably-sized set of probes and to increase the likelihood of detecting biologically significant changes in methylation levels. HpaII-amplifiable fragments on the HELP microarray were annotated to the nearest gene up to a maximum distance of 5 kilobases from the transcription start site. The networks and functional analyses were generated through the use of Ingenuity Pathway Analysis (IPA, Ingenuity Systems, http://www.ingenuity.com/products/pathways_analysis.html).

### Quantitative real time polymerase chain reaction (RT-PCR)

RNA was extracted from cells using RNeasy Mini Kit (Qiagen) according to the manufacturer's protocols. cDNA was prepared using SuperScript® VILO cDNA Synthesis kit (Life Technologies) and detected by SsoFast™ EvaGreen® (BioRad) on an BioRad CFX96 thermal cycler (BioRad). Gene expression was normalized to hypoxanthine phosphoribosyltransferase (HPRT) and expressed relative to untreated control using the ΔΔCT method. Thermal cycler conditions were: initial step of 30 sec at 95°C followed by 40 cycles of 5 sec at 95°C (denature) and 5 sec at 60°C (anneal/extend). For primers, see Supplementary Table 6.

### Western blot analysis

Cells were lysed in modified RIPA buffer containing 50 mM Tris-HCl (pH = 8.0), 300 mM NaCl, 10% NP-40, 1% sodium deoxycholate, and 0.1% SDS, and a protease inhibitor cocktail tablet (Roche Applied Science). Protein extracts, approximately 30 μg of each sample, were resolved by SDS-PAGE followed by immunoblotting with Noxa antibody (114C307, mouse monoclonal antibody, EMD Millipore), DNMT1 (H-300, rabbit polyclonal, Santa Cruz Biotechnologies), and actin antibody (C-11, horseradish peroxidase (HRP), goat polyclonal antibody; Santa Cruz Biotechnologies), and detected by enhanced chemiluminescence (ECL; Santa Cruz Biotechnology). After treatment, cells were harvested and washed with ice-cold PBS, and subsequently lysed with RIPA buffer with fresh protease inhibitors. Blot patterns were analyzed using Image-J software (http://rsbweb.nih.gov/ij/), providing a quantitative measure of protein expression.

### *In vivo* tumor models

All animal studies were carried out in accordance with the guidelines of the Institutional Animal Care and Use Committee of the Icahn School of Medicine at Mount Sinai. Four-five-week-old Nude-*Foxn1^nu^* athymic nude female mice were purchased from Harlan Laboratories. Five million of Z138, MINO, and Rec-1 cells were mixed with Matrigel (BD Biosciences) in 1:1 ratio and injected subcutaneously into the right and left flanks of each mouse. Tumors measuring 0.5 cm in diameter appeared approximately in 10–20 days post injection of cancer cells. When the tumors approached 40–50 mm^3^, the mice were divided into 4 groups of 4 mice: (i) control group, which received saline with 0.02% dimethyl sulfoxide (DMSO) by subcutaneous injections every week for 3 weeks; (ii) DAC alone group, which received a dose of 1.2 mg/kg given by i.p. and divided into 6 equal doses on days 1, 3, 5, 8, 10, 12 (0.2 mg/kg each); (iii) BZM alone, which received a dose of 100 μg/kg (for BZM-resistant MINO and Rec-1 cells) and 75 μg/kg (for BZM-sensitive Z138 cells) subcutaneously every week for 3 weeks; (iv) combination group, which received BZM and DAC at a doses described above. The data were expressed as average tumor volume (mm^3^) per group as a function of time. Tumors volume and mouse weight were assessed three times per week.

## SUPPLEMENTARY FIGURES AND TABLES




