# Supplementary material for: Harnessing Noxa demethylation to overcome Bortezomib resistance in mantle cell lymphoma
Source: Oncotarget. 2015 Feb 25;6(29):27332–42. doi: 10.18632/oncotarget.2903 (PMC4694993; doi:10.18632/oncotarget.2903)
Supplement: Supplementary file 1 [file oncotarget-06-27332-s001.pdf]

SUPPLEMENTARY FIGURES AND TABLES

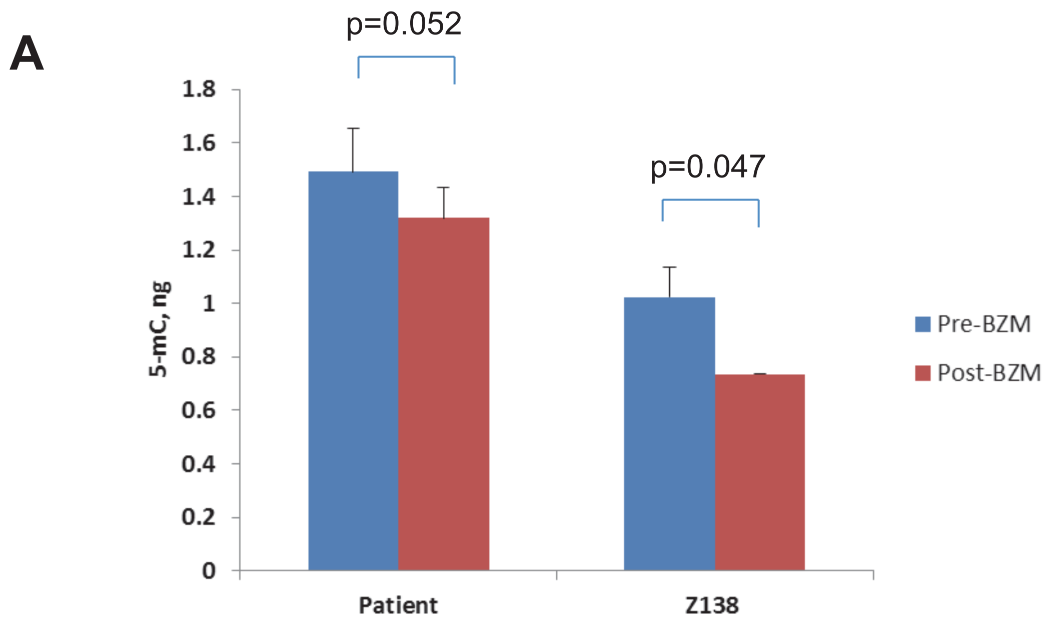

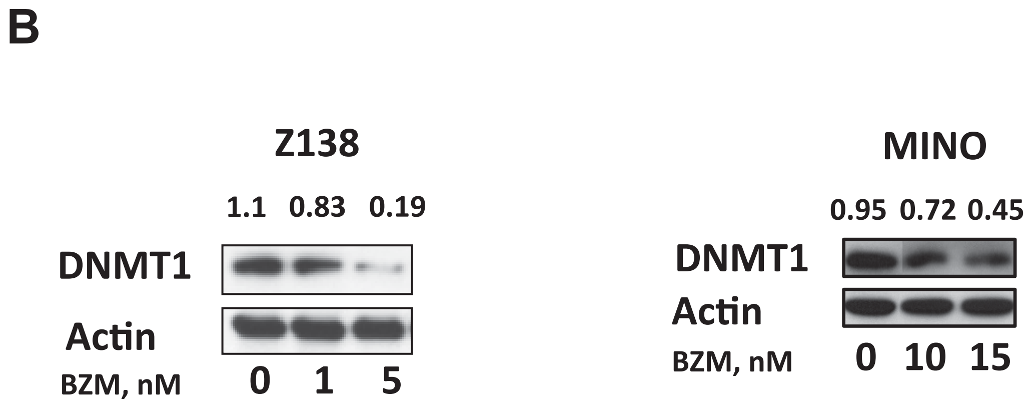

Supplementary Figure S1: BZM causes DNA hypomethylation and affects DNMT1 expression in tumor cells from MCL patients and cell lines. **(A)** Global DNA Methylation measured by 5-methylcytosine-modified genomic DNA quantification in MCL patient sample (average of two MCL patient samples) and Z138 MCL cell line before and 24 hours after BZM treatment. **(B)** Z138 and MINO cells were exposed to the indicated doses of BZM for 24 hours. Whole cell lysates were obtained and DNMT1 protein levels were analyzed by Western blotting. The ratio of DNMT1 to actin was determined by densitometry using ImageJ.

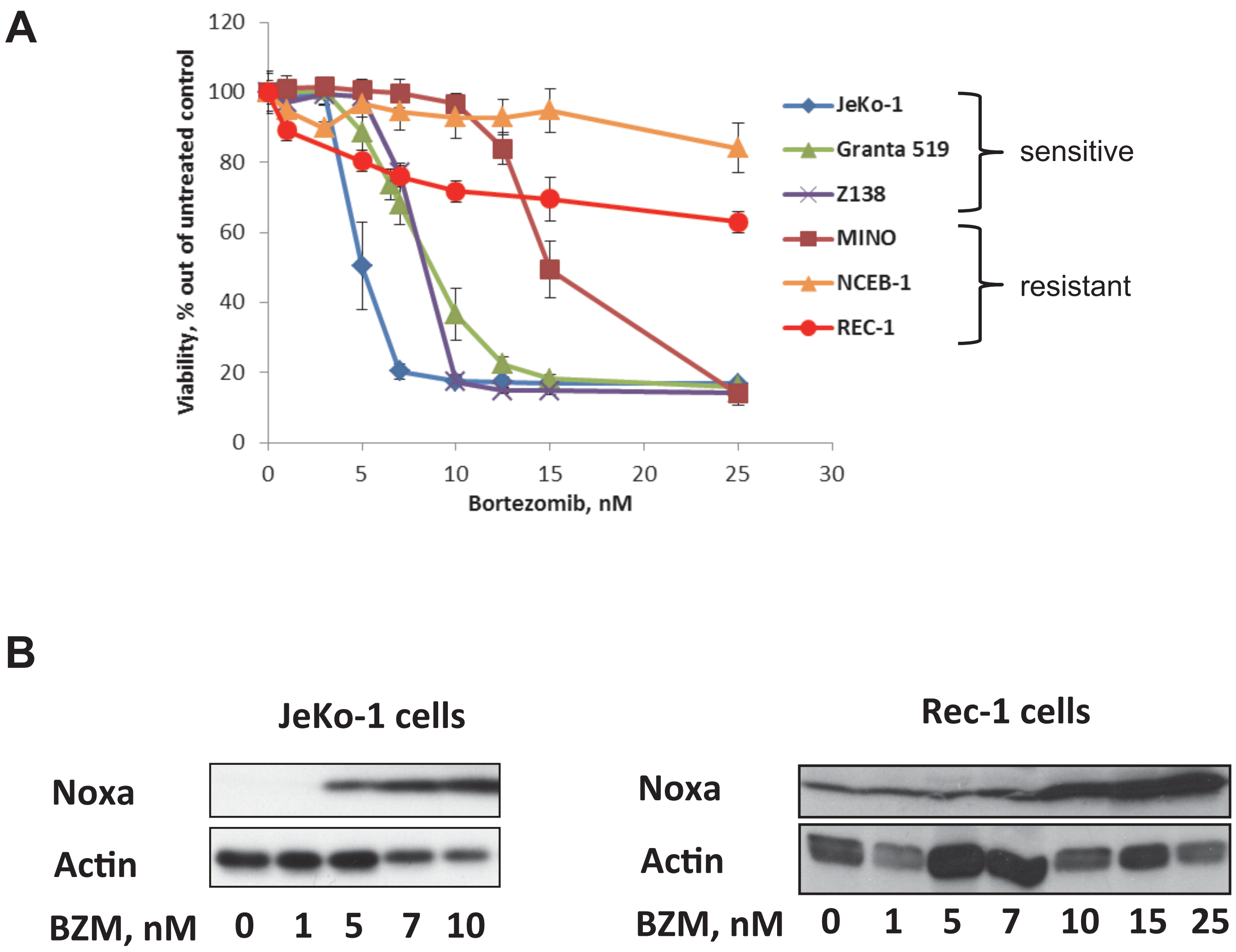

Supplementary Figure S2: BZM induced growth inhibition and Noxa protein expression in MCL cells. (A) 10^6 MCL cells/ml was treated with BZM at concentrations of 5–25 nMM for 48 hours. Cell viability is expressed as percentage of control. Jeko-1, Granta 519, and Z138 represent sensitive lines while Mino, NCEB-1, and REC-1 represent resistant lines. Data represents average of 8 replicates per condition in two independent experiments; bars represent standard deviation. **(B)** Noxa protein expression in JeKo-1 and Rec-1 MCL cells treated with increasing concentrations of BZM for 24 hours and detected by Western blotting analysis. Endogenous Actin was used as loading control.

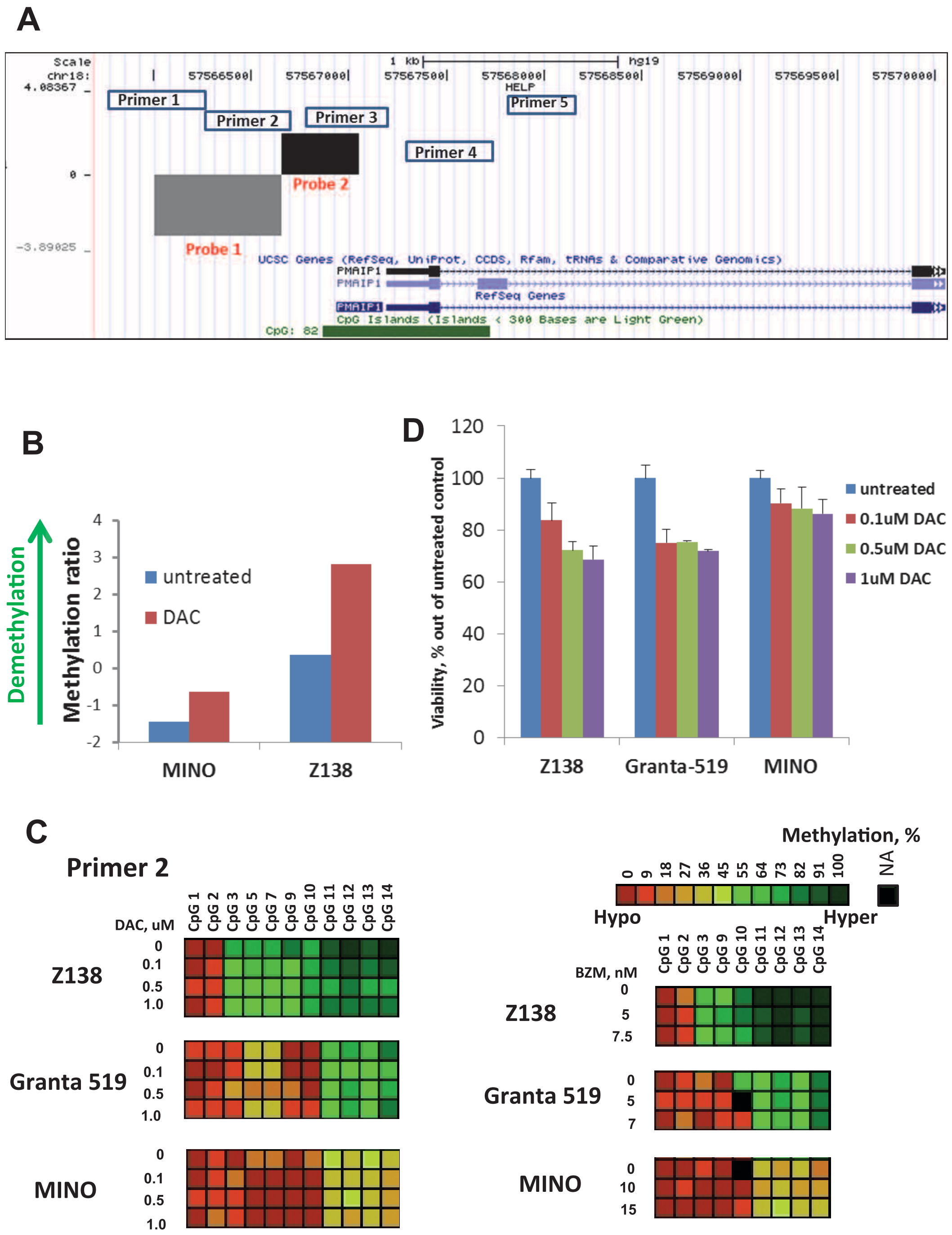

Supplementary Figure S3: DAC and BZM cause Noxa promoter demethylation in MCL cell lines. (A) University of California Santa Cruz (UCSC) Browser view of the Noxa (PMAIP1) locus. The areas covered by the HELP array Noxa Probe 1 (grey square) and Probe 2 (black square), the MassArray primers 1–5, a CpG island, direction of transcription, and Noxa (PMAIP1) gene start are indicated in the USCS Browser view. **(B)** Changes in methylation values estimated by HpaII/MspI ratios difference (Y-axis) in Noxa promoter HELP array probes in MCL cell lines after treatment with DAC using HELP array. **(C)** Methylation of CpGs covered by primer 2 after treatments with BZM and DAC measured by MassArray. Scale shows percentage of methylation from 0% (low, in dark orange) to 100% (high, in dark green) for each CpGs. **(D)** Viability of MCL cell lines after 72 hours of treatment with DAC three times every 24 hr. Error bars indicate SD.

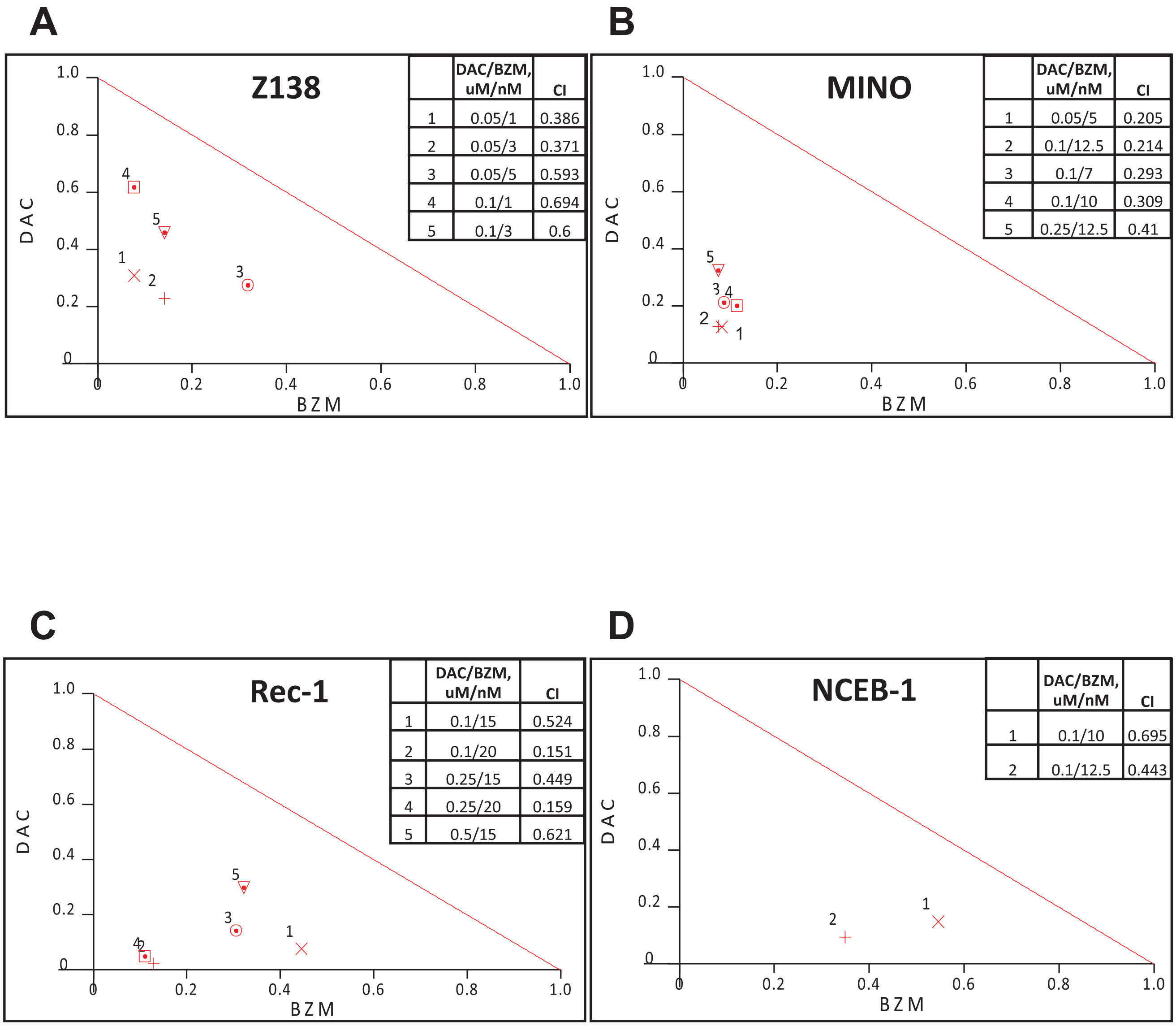

A – Z138

	DAC/BZM, uM/nM	CI
1	0.05/1	0.386
2	0.05/3	0.371
3	0.05/5	0.593
4	0.1/1	0.694
5	0.1/3	0.6

B – MINO

	DAC/BZM, uM/nM	CI
1	0.05/5	0.205
2	0.1/12.5	0.214
3	0.1/7	0.293
4	0.1/10	0.309
5	0.25/12.5	0.41

C – Rec-1

	DAC/BZM, uM/nM	CI
1	0.1/15	0.524
2	0.1/20	0.151
3	0.25/15	0.449
4	0.25/20	0.159
5	0.5/15	0.621

D – NCEB-1

	DAC/BZM, uM/nM	CI
1	0.1/10	0.695
2	0.1/12.5	0.443

Supplementary Figure S4: DAC synergies with BZM in MCL *in vitro*. (A–D) DAC sensitizes BZM-sensitive and BZM-resistant MCL cells to BZM *in vitro* in Z138, MINO, Rec-1, and NCEB-1 cells, respectively. Combination indices (CI) values were determined using the Chou-Talalay equation, as calculated by Calcusyn software.

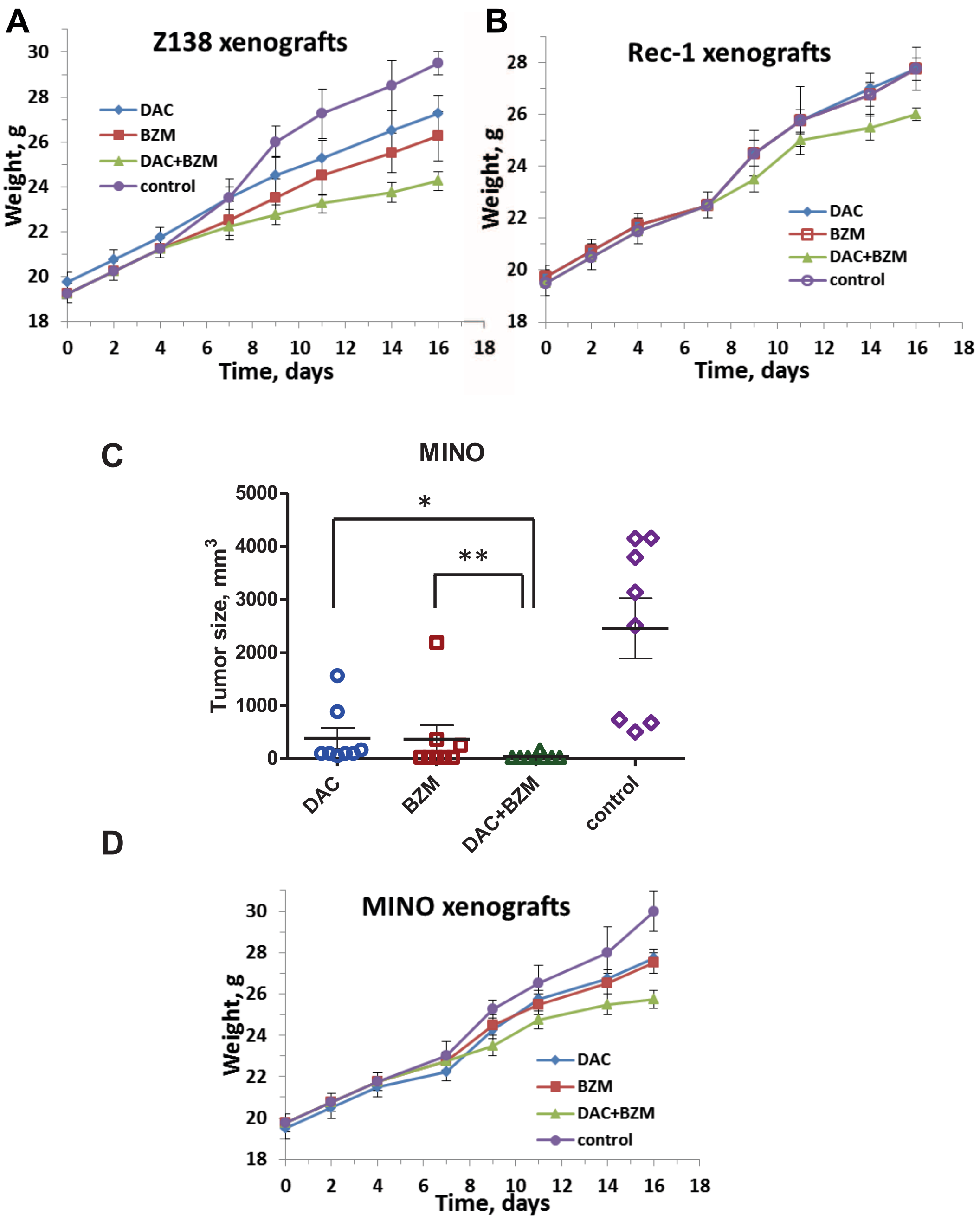

Supplementary Figure S5: The effect of BZM and DAC in MCL xenograft experiments. (A–B, D) Mouse weight for treatment cohorts expressed as means. Z138, Rec-1, and MINO MCL cell line were used. DAC was given IP as 3 injections of 0.2 mg/kg on day 1, 3, 5, 8, 10, 12 (1.2 mg/kg total), BZM was injected SC, 1 injection of 75 µg/kg (Z138) or 100 µg/kg (Rec-1, MINO) per week during 3 weeks. Control group of mice received SC diluent control (0.01% DMSO) injection once per week during 3 weeks. **(C)** BZM and DAC induce tumor growth inhibition in the MINO xenograft models. Tumor volumes for treatment cohorts expressed as means +/– SEM in 16 days from the beginning of treatment. DAC was given IP as 6 injections of 0.2 mg/kg on day 1, 3, 5, 8, 10, 12 (1.2 mg/kg total), BZM was injected SC, 1 injection of 100 µg/kg per week during 3 weeks. Control group of mice received SC diluent control (0.01% DMSO) injection once per week during 3 weeks. $N = 8$ for each group of mice. Statistical significance between the groups is indicated as follows:*$P = 0.1$ for DAC+BZM vs DAC alone; **$P = 0.24$ for DAC+BZM vs BZM alone.

Supplementary Table 1: Hypomethylated genes in MCL Patients after BZM treatment (13102 Refseq IDs, 9561 genes, DM < 0.05, $p < 0.05$)

Supplementary Table 2: GSEA categorizes hypomethylated genes in MCL Patients after BZM treatment by gene families

Gene family	Number of genes
Tumor suppressors	52
Oncogenes	197
Translocated cancer genes	177
Protein kinases	283
Cell differentiation markers	158
Homeodomain proteins	158
Transcription factors	905
Cytokines and growth factors	188

Supplementary Table 3: Top canonical pathways categorized by the Ingenuity pathway analysis among hypomethylated genes in MCL patient samples after BZM treatment

Ingenuity Canonical Pathways	*p*-value	Ratio (Number of molecules in experimental dataset out of total number in the pathway set)
Molecular Mechanisms of Cancer	0.000000085	198/379(0.522)
Axonal Guidance Signaling	0.000000104	224/433(0.517)
Purine Metabolism	0.000000443	160/398(0.402)
Wnt/β-catenin Signaling	0.000000521	107/174(0.615)
Protein Ubiquitination Pathway	0.000000762	154/274(0.562)
Cell Cycle: G1/S Checkpoint Regulation	0.00000251	42/61(0.689)
PI3K/AKT Signaling	0.00000442	77/140(0.55)
ERK5 Signaling	0.000005	45/64(0.703)
Protein Kinase A Signaling	0.00000529	176/331(0.532)
Glucocorticoid Receptor Signaling	0.00000625	151/295(0.512)
Cell Cycle: G2/M DNA Damage Checkpoint Regulation	0.00000764	34/49(0.694)
Cardiac Hypertrophy Signaling	0.0000225	129/245(0.527)
PTEN Signaling	0.0000256	69/124(0.556)
Cyclins and Cell Cycle Regulation	0.0000303	53/89(0.596)
Small Cell Lung Cancer Signaling	0.0000526	48/89(0.539)
Notch Signaling	0.0000536	29/43(0.674)
Ephrin Receptor Signaling	0.05051	102/200(0.51)
Assembly of RNA Polymerase II Complex	0.0000588	36/56(0.643)
Role of Osteoblasts, Osteoclasts and Chondrocytes in Rheumatoid Arthritis	0.0000689	125/240(0.521)
ATM Signaling	0.0000705	37/54(0.685)

Supplementary Table 4: Functional groups enriched by the Ingenuity pathway analysis of hypomethylated genes in MCL Patients after BZM treatment

in TMZ resistant DLBCL cell lines	Diseases or Functions Annotation	*p*-Value	# Molecules
Gene Expression	transcription	3.59E-57	1371
Gene Expression	transcription of RNA	2.17E-55	1343
Gene Expression	expression of RNA	3.58E-54	1500
Cellular Growth and Proliferation	proliferation of cells	1.01E-52	2488
Cell Death and Survival	cell death	1.94E-50	2324
Organismal Survival	organismal death	5.02E-48	1715
Gene Expression	expression of DNA	5.55E-45	1097
Gene Expression	transcription of DNA	1.71E-44	1047
Cell Death and Survival	apoptosis	3.01E-44	1860
Cell Death and Survival	necrosis	1.01E-40	1796
Cancer	Cancer	1.13E-37	4552
Gene Expression	activation of DNA endogenous promoter	1.46E-36	751
Cell Death and Survival	cell death of tumor cell lines	2.15E-34	1069
Tissue Morphology	abnormal morphology of embryonic tissue	3.83E-32	468

Supplementary Table 5: Statistically significant differences in methylation of Bcl-2 family members in MCL patient samples before and after BZM treatment

Array Probe ID	Refseq ID	Gene Symbol	Entrez Gene Name	*P* value*
MSPI0406S00569367	NM_004322, NM_032989	BAD	BCL2-associated agonist of cell death	0.0163
MSPI0406S00569368	NM_004322, NM_032990	BAD	BCL2-associated agonist of cell death	0.0005
MSPI0406S00569369	NM_004322, NM_032991	BAD	BCL2-associated agonist of cell death	0.0160
MSPI0406S00317467	NM_001188	BAK1	BCL2-antagonist/killer 1	0.0006
MSPI0406S00898897	NM_014417	BBC3 (Puma)	BCL2 binding component 3	0.0106
MSPI0406S00898898	NM_014417	BBC3 (Puma)	BCL2 binding component 3	0.0342
MSPI0406S00125137	NM_006538, NM_138621, NM_207002	BCL2L11 (BIM)	BCL2-like 11 (apoptosis facilitator)	0.0025
MSPI0406S00125138	NM_006538, NM_138621, NM_207002	BCL2L11 (BIM)	BCL2-like 11 (apoptosis facilitator)	0.0169
MSPI0406S00954989	NM_001196, NM_197966, NM_197967	BID	BH3 interacting domain death agonist	0.0038
MSPI0406S00852330	NM_021127	PMAIP1 (Noxa)	phorbol-12-myristate-13-acetate-induced protein 1	0.0395
MSPI0406S00852331	NM_021127	PMAIP1 (Noxa)	phorbol-12-myristate-13-acetate-induced protein 1	0.0182

**P* values are calculated for the methylation differences between samples before and after BZM treatment for loci representing Bcl-2 family member genes.

Analysis was performed for the samples from 6 MCL patients. All *P*-values displayed were calculated by one-sided Student's *t*-test.

Supplementary Table 6: Primer sequences for siRNA knockout, MassARRAY, and Quantitative Real-Time Polymerase Chain Reaction

Primer Name	Primer Sequence
**siRNA knockout**	
Noxa (PMAIP1) siRNA, # 05	5′ AAACUGAACUUCCGGCAGA
Noxa (PMAIP1) siRNA, # 08	5′ GCAAGAACGCUCAACCGAG
Non-targeting siRNA	5′ UAAGGCUAUGAAGAGAUAC
**MassARRAY**	
PMAIP1 Primer 1 Forward	5′ aggaagagagTATTTAGGTAGAGAGTATTGTTTTGATTT
PMAIP1 Primer 1 Reverse	5′ cag**taatacgactcactatagggaga**aggctCCAAACTAAACACTTCTAAAAAACCC
PMAIP1 Primer 2 Forward	5′ aggaagagagAGGAGATGTTAGAGGTTTTGTGAGA
PMAIP1 Primer 2 Reverse	5′ cag**taatacgactcactatagggaga**aggctTCAAAACAATACTCTCTACCTAAATAAAC
PMAIP1 Primer 3 Forward	5′ aggaagagagTGTTTAGTGAGAATTTTTTTGGTGA
PMAIP1 Primer 3 Reverse	5′ cag**taatacgactcactatagggaga**aggctCCAATCTCTTTTCTAAACTTATTTACCC
PMAIP1 Primer 4 Forward	5′ aggaagagagAGGAGGAAAGGAGTTTTTTGTTTT
PMAIP1 Primer 4 Reverse	5′ cag**taatacgactcactatagggaga**aggctCCAACATCCCTACCTACAAAACTATT
PMAIP1 Primer 5 Forward	5′ aggaagagagGGTTGTTTTTAGGAGAAAGTTATTG
PMAIP1 Primer 5 Reverse	5′ cag**taatacgactcactatagggaga**aggctACAACAACAATATAACCCACACAAA
**Quantitative Real-Time Polymerase Chain Reaction**	
PMAIP1 Forward	5′ AGCTGGAAGTCGAGTGTGCT
PMAIP1 Reverse	5′ TCCTGAGCAGAAGAGTTTGGA
HPRT Forward	5′ AAAGGACCCCACGAAGTGTT
HPRT Reverse	5′ TCAAGGGCATATCCTACAACAA
